# Social patterns and differentials in the fertility transition in the context of HIV/AIDS: evidence from population surveillance, rural South Africa, 1993 – 2013

**DOI:** 10.1186/s12963-016-0079-z

**Published:** 2016-03-25

**Authors:** Brian Houle, Athena Pantazis, Chodziwadziwa Kabudula, Stephen Tollman, Samuel J. Clark

**Affiliations:** School of Demography, The Australian National University, #9 Fellows Road, Acton, ACT Australia, Canberra, Australia; Institute of Behavioral Science, University of Colorado at Boulder, Boulder, CO USA; MRC/Wits Rural Public Health and Health Transitions Research Unit (Agincourt), School of Public Health, Faculty of Health Sciences, University of the Witwatersrand, Johannesburg, South Africa; Department of Sociology, University of Washington, Seattle, WA USA; School of Public Health, Faculty of Health Sciences, University of the Witwatersrand, Johannesburg, South Africa; INDEPTH Network, Accra, Ghana; Centre for Global Health Research, Umeå University, Umeå, Sweden; Department of Population Health, London School of Hygiene and Tropical Medicine, London, UK

**Keywords:** Fertility, South Africa, Socio-economic status, Mozambican refugee, Discrete time event history analysis

## Abstract

**Background:**

Literature is limited on the effects of high prevalence HIV on fertility in the absence of treatment, and the effects of the introduction of sustained access to antiretroviral therapy (ART) on fertility. We summarize fertility patterns in rural northeast South Africa over 21 years during dynamic social and epidemiological change.

**Methods:**

We use data for females aged 15–49 from the Agincourt health and socio-demographic surveillance system (1993–2013). We use discrete time event history analysis to summarize patterns in the probability of any birth.

**Results:**

Overall fertility declined in 2001–2003, increased in 2004–2011, and then declined in 2012–2013. South Africans showed a similar pattern. Mozambicans showed a different pattern, with strong declines prior to 2003 before stalling during 2004–2007, and then continued fertility decline afterwards. There was an inverse gradient between fertility levels and household socioeconomic status. The gradient did not vary by time or nationality.

**Conclusions:**

The fertility transition in rural South Africa shows a pattern of decline until the height of the HIV/AIDS pandemic, with a resulting stall until further decline in the context of ART rollout. Fertility patterns are not homogenous among groups.

## Background

By the 1990s, South Africa was well advanced in its transition to low fertility, having halved fertility between the 1960s and 1990s. Up until the 1980s, fertility decline was gradual [1]. Fertility decline quickly resumed for the general population by the early 1990s and continued through 2004. At the national level in South Africa, fertility continued to decline to an estimated total fertility rate (TFR) of 2.57 in 2014 [2], while in neighboring Mozambique fertility has remained much higher, with a TFR of 5.5 in 2003, increasing to 5.9 as measured in 2011 [3]. In Agincourt, a rural area in Mpumalanga province, South Africa, earlier work by Garenne et al. [4] has found that fertility decline stalled in the 1980s with the influx of Mozambican refugees who arrived in large numbers and had higher fertility than the resident South Africans. Another stall in fertility decline was also detected in the early 2000s and had again been thought to be attributable to the higher fertility of Mozambican immigrants, though Ibisomi et al. [5] find that during this period it was South African fertility that increased while Mozambican fertility declined.

Socioeconomic development and household socioeconomic status has long been hypothesized to contribute to fertility decline though evidence in sub-Saharan Africa (SSA) has been inconsistent, and it has been difficult to identify levels of socioeconomic development or affluence that are associated with initiation of decline or that trigger stalls in fertility decline [6–8]. At the household level, higher socioeconomic status (SES) has been found to be generally associated with lower desired family size, lower completed fertility, and greater use of contraception in many African contexts [9–11]. In South Africa, women born in the 1980s have on average two fewer children than women born in the 1950s through 1970s, with about half of the difference attributable to better schooling, decreasing marriage rate, and growing income [12]. In Agincourt, Williams et al. [13] found an increasing convergence in education and wealth indicators between Mozambican and South African women, and suggested that increased access to education contributed to fertility declines among Mozambicans.

HIV has been theorized to lower fertility through several biological and social mechanisms; for example: reduced coital frequency; sexually transmitted infection (STI) co-infection; delayed onset of sexual debut and/or first union; reduced pre-marital sex and re-marriage; increased spousal separation and marriage dissolution; increased condom use; increased post-partum amenorrhea; reduced pregnancy rates and increased fetal loss; and reduced sperm production. HIV has also been theorized to increase fertility through other mechanisms, such as reduced breastfeeding, reduced post-partum abstinence, and child replacement due to increased infant mortality [14]. Evidence from some SSA contexts indicate a downward effect of HIV on fertility [14–16], while other work suggests a connection between HIV-related increases in mortality and stalls in fertility decline [17]. HIV is generally associated with female subfecundity, and in particular among women in their late 20s and older, related to duration of HIV infection [18]. Terceira et al. [18] found much lower fertility among HIV positive than HIV negative women in Zimbabwe and attributed HIV to one-quarter of fertility decline since the 1980s. In South Africa it is generally suggested that HIV is a major contributor to the most recent fertility decline. Burger et al. [12] suggest that it is likely one of the main unobserved factors contributing to the decline in fertility seen in their study. Garenne et al. [4] working with the Agincourt data found that increasing HIV mortality (and declining survival of women) was reducing net reproductive rates. Studies conducted in South Africa looking at contraception use (a proximate determinant of fertility) amongst HIV positive and HIV negative women have found that HIV positive women are more likely to use condoms and dual contraception than HIV negative women up to 14 months after a birth [19].

Earlier work on the effects of HIV on fertility in South Africa have stressed the important role of antiretroviral treatment (ART) roll-out in shaping both the HIV epidemic and fertility [4]. Qualitative work with people living with HIV (PLHIV) in Cape Town, South Africa has indicated that the availability of ART would likely affect fertility decisions [20]. Research looking at the effects of ART on fertility has found the following: an increase in fertility desire but no increase in fertility in Uganda [21]; higher fertility after 4 years of follow-up for women on ART compared to women not yet on ART in a cross-national analysis of prevention of mother to child transmission (PMTCT) program data from seven SSA countries [22]; longer duration on ART was associated with higher fertility desires in a cross-sectional study of women in ART treatment in South Africa [23].

We aim to summarize overall patterns in fertility in rural South Africa over 21 years during dynamic social and epidemiological change, including the emergence of the HIV pandemic and the rollout of ART. We use a longitudinal, robust population surveillance dataset and analyze trends using a unified, statistical framework that permits detailed hypothesis testing of differences and incorporation of uncertainty in our estimates. Finally, we examine variation in fertility patterns according to social groups, including nationality and household socioeconomic status.

This study will further the literature on fertility in SSA in several ways. First, the long period of follow-up allows for the analysis of trends over a period of two decades, shedding light on how immigrant fertility assimilates to host population fertility, the robustness of SES effects on fertility over time, the effects of high prevalence HIV on fertility in the absence of treatment, and the effects of the introduction of sustained access to ART on fertility in a high HIV prevalence setting. Literature investigating these relationships has largely been limited to short periods of follow-up or cross-sectional analyses and thus unable to observe how these effects behave over time within a community. Second, previous methods used from Agincourt on fertility were direct calculation of rates (or decomposition of those rates) and did not supply confidence intervals [4, 5]. Using discrete time event history analysis, we are able to quantify uncertainty around our estimates as well as incorporate multiple covariates for fertility and test their interactions. Third, looking at the role of HIV and ART on fertility, a key strength of our study is that we are analyzing fertility at the population level. A frequent weakness of studies on fertility and HIV is that they are restricted only to people living with HIV without a comparison group or being situated in the larger population fertility trends. Consequently, they are only able to look at individual level fertility preferences or fertility outcomes, rather than how HIV and ART may be affecting overall population fertility.

## Methods

### Setting and data

We use household data collected from 1993–2013 by the Agincourt Health and Demographic Surveillance System (AHDSS) that has been tracking the population living in the Agincourt subdistrict of Bushbuckridge, Mpumalanga province, South Africa [24]. Annually, fieldworkers have collected information from the most knowledgeable person in each household, with systematic collection of vital events (e.g., births, deaths), migration, household socioeconomic indicators, and other individual and household information. The population under surveillance in 2011 was approximately 90,000 people residing in 16,000 households, while in 1994 the population was approximately 66,000 [24]. The rural area, located in northeast South Africa near the Mozambique border, suffers from limited infrastructure and employment opportunities. Until recently, mortality has been increasing among children, young and middle-aged adults, largely due to the HIV epidemic [25–27]. ART became available in the study area in 2007 [28].

Over 30 % of the population comprises Mozambican immigrants, formerly refugees who entered the area during/following the Mozambican civil war [24]. We code nationality status as the respondent being either South African or Mozambican. There were not enough other nationalities to include as a separate category.

Household socioeconomic status (SES) has been measured biannually since 2001 using a validated, 34-item asset index [29]. We calculate an overall asset score for each household as in Houle et al. [26]. We code household SES by taking tertiles of this absolute SES asset scale pooled across all years.

We categorize time periods to both detect change along the dimensions of our statistical models and contextualize the dynamics of the HIV epidemic in the study population over time. Most time periods include 4-year intervals to simultaneously capture temporal change as well as sufficient births in each cell. Similarly, we categorize female ages of 15–49 years into standard 5-year age-groups to both detect discrete change as well as to facilitate comparisons with other demographic studies.

### Analysis

As SES was measured biannually since 2001, we impute missing values from 2001–2013 using partial mean matching (based on nearest two neighbors) with five imputations. Because of the multilevel nature of the data, we follow the recommendations of Gelman, Hill [30] and derive a household-year level data set for imputation which includes within each household-year aggregated form of individual-level measurements, including: counts of males, females, Mozambicans, and South Africans, ages under 20, 20–59, and 60+, and 1–2 year lags of household SES to account for patterns in SES over time. Diagnostics of the imputation results compare the distribution of imputed values to the observed finding that imputed household SES tracked well with the observed values. Time series plots of observed and imputed values within households were also compared and found plausible.

We model fertility using discrete time event history analysis for repeating events [31]. We organize data as person-years, including one record for each fully observed person-year lived by each female aged 15–49 years. We set the values of covariates at the beginning of the person-year. We begin with a bivariate logistic regression to explore time trends using multilevel, logistic regression including a random intercept for the individual and indicators (dummy variables) for year. We next model the yearly probability of any birth using multilevel, logistic regression including a random intercept for the individual and include covariates: age, time period, nationality, and SES. Because of the limited time span of SES data (2001 onwards), and due to the small proportion of nationalities other than South African and Mozambican, we estimate four models: (1) fertility by age and time; (2) fertility by age, time, and nationality; (3) fertility by age, time, and SES; and (4) fertility by age, time, nationality, and SES. When including SES we use inference according to Rubin [32] to account for variance between and within imputations. We summarize our models using predictive probabilities for discrete groups.

We also test a model of multiple births using multilevel, multinomial logistic regression including a random intercept for the individual and covariates of age and time period. Given the rarity of triplets, we categorize the outcome into no birth, single, and multiple births. The predictions from the multiple birth outcomes were unstable (likely due to small *n* = 285) and are not presented. All analyses were completed using Stata 13 [33].

### Ethics, consent and permissions

The Agincourt health and socio-demographic surveillance system (HDSS) was reviewed and approved by the Committee for Research on Human Subjects (Medical) of the University of the Witwatersrand (protocol M960720 and M081145). A record of participant consent is kept of the household respondent who consented to the interview.

## Results

**Fig. 1:**
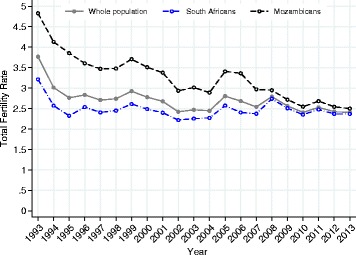
Total fertility rate (TFR) by year, overall and by nationality, for females ages 15–49, Agincourt health and demographic surveillance system, South Africa, 1993–2013

**Fig. 2:**
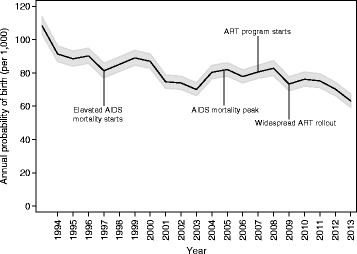
Annual probability of any birth by year for females ages 15–49, Agincourt health and demographic surveillance system, South Africa, 1993–2013. Multilevel model includes a random intercept for the individual

Table 1 presents descriptive characteristics of the estimation sample by time period. Figure 1 presents the total fertility rate (TFR) by year, for the whole study area population and by nationality. In 1993, the TFR was 3.77 overall (4.83 for Mozambicans and 3.21 for South Africans), falling to 2.40 overall in 2013 (2.50 for Mozambicans and 2.37 for South Africans). Figure 2 presents predicted annual probabilities of birth per 1000 by year from bivariate logistic regression. Figures 1 and 2 show similar time trends in period fertility, with Fig. 1 based on the TFR while Fig. 2 uses model-based probabilities. For women aged 15–49, both the TFR and the predicted probability of giving birth are weighted averages of age-specific fertility rates for the TFR and age-specific probabilities of giving birth for the predicted probability of giving birth. By construction the TFR gives each age group equal weight, whereas the age-specific probabilities of giving birth are effectively weighted by the fraction of women in each age group, i.e., the age distribution of women in the age range 15–49. Together with the inherent scale difference between rates and probabilities and the purposeful rescaling of the TFR so that it can be interpreted as the number of births that a woman would have living through the associated age-specific fertility rates, the differences in the construction of the averages yields numbers that are different and trends that are similar but slightly different, reflecting the evolution of the age structure of the female population.

**Table 1:** Counts of person-years, births, and nationality groups by time period for females ages 15–49, Agincourt health and demographic surveillance system, South Africa

Time period	Person years	Births	South Africans^a^	Mozambicans^a^
1993–1996	59,236	5750	42,015	16,808
1997–2000	63,667	5700	45,556	18,019
2001–2003	50,889	3900	36,563	14,315
2004–2007	73,244	6199	52,351	20,854
2008–2011	60,295	4894	41,817	18,404
2012–2013	30,406	2154	21,404	8941

^a^counts do not sum to person years due to other nationalities (0.2 % of person-years)

Figure 2 is annotated to indicate the times when HIV/AIDS-related events took place. AIDS-related mortality first became noticeable around 1997 and peaked in 2005. The ART delivery program started slowly in 2007 and achieved widespread effectiveness around 2009. The annual probability of giving birth age 15–49 does not change dramatically during the periods of time affected by HIV and ART. It is possible that fertility decline slowed slightly as HIV began to affect mortality and resumed a faster decline after ART became widely available, but because we do not have individual-level data on HIV or ART status, we cannot rigorously test this hypothesis.

### Fertility patterns by age and time

To explore overall fertility patterns we estimated a model including age and time; an interaction between age and time period significantly improved model fit (*p* < 0.001) and resulted in the final model. Estimation results are presented in model M1 (Table 2).

**Table 2:** Logistic regression on birth by age, time, and nationality, females ages 15–49, Agincourt health and demographic surveillance system, South Africa

	Model M1			Model M2		
	Odds ratio	95 % CI	*p*-value	Odds ratio	95 % CI	*p*-value
Mother's age						
15–19	1.000	[1.000, 1.000]	.	1.000	[1.000, 1.000]	.
20–24	1.276	[1.177, 1.384]	<0.001	1.173	[1.078, 1.277]	< 0.001
25–29	1.317	[1.211, 1.433]	< 0.001	1.189	[1.088, 1.299]	< 0.001
30–34	1.228	[1.123, 1.342]	< 0.001	1.128	[1.027, 1.240]	0.012
35–39	1.013	[0.915, 1.121]	0.809	0.926	[0.831, 1.031]	0.159
40–44	0.489	[0.427, 0.562]	< 0.001	0.404	[0.348, 0.469]	< 0.001
45–49	0.230	[0.184, 0.286]	< 0.001	0.140	[0.107, 0.183]	< 0.001
Time period						
1993–1996	1.000	[1.000, 1.000]	.	1.000	[1.000, 1.000]	.
1997–2000	0.926	[0.853, 1.005]	0.065	0.961	[0.882, 1.046]	0.357
2001–2003	0.806	[0.737, 0.882]	< 0.001	0.842	[0.767, 0.924]	< 0.001
2004–2007	0.926	[0.856, 1.002]	0.056	0.982	[0.904, 1.067]	0.674
2008–2011	0.880	[0.807, 0.958]	0.003	1.001	[0.914, 1.095]	0.99
2012–2013	0.786	[0.701, 0.880]	< 0.001	0.890	[0.789, 1.003]	0.056
Mother's age X time period						
20–24 X 1997–2000	1.004	[0.895, 1.126]	0.951	1.006	[0.897, 1.128]	0.918
20–24 X 2001–2003	1.025	[0.904, 1.162]	0.704	1.031	[0.909, 1.168]	0.636
20–24 X 2004–2007	0.965	[0.863, 1.078]	0.523	0.982	[0.879, 1.097]	0.747
20–24 X 2008–2011	1.030	[0.916, 1.159]	0.62	1.029	[0.915, 1.157]	0.636
20–24 X 2012–2013	1.056	[0.906, 1.230]	0.487	1.056	[0.906, 1.230]	0.488
25–29 X 1997–2000	1.032	[0.916, 1.162]	0.604	1.046	[0.929, 1.178]	0.454
25–29 X 2001–2003	1.018	[0.894, 1.160]	0.788	1.036	[0.910, 1.180]	0.595
25–29 X 2004–2007	0.992	[0.884, 1.114]	0.893	1.016	[0.906, 1.140]	0.782
25–29 X 2008–2011	0.965	[0.853, 1.092]	0.573	0.983	[0.869, 1.112]	0.782
25–29 X 2012–2013	0.899	[0.766, 1.055]	0.193	0.919	[0.783, 1.078]	0.299
30–34 X 1997–2000	1.000	[0.882, 1.135]	0.998	0.998	[0.880, 1.132]	0.974
30–34 X 2001–2003	0.901	[0.783, 1.037]	0.147	0.902	[0.784, 1.038]	0.15
30–34 X 2004–2007	0.927	[0.819, 1.050]	0.232	0.940	[0.830, 1.063]	0.323
30–34 X 2008–2011	0.923	[0.809, 1.054]	0.238	0.928	[0.813, 1.059]	0.267
30–34 X 2012–2013	0.875	[0.737, 1.039]	0.127	0.877	[0.739, 1.041]	0.135
35–39 X 1997–2000	0.882	[0.764, 1.018]	0.087	0.897	[0.777, 1.036]	0.141
35–39 X 2001–2003	0.871	[0.744, 1.020]	0.086	0.891	[0.761, 1.043]	0.151
35–39 X 2004–2007	0.819	[0.711, 0.943]	0.005	0.836	[0.726, 0.962]	0.012
35–39 X 2008–2011	0.779	[0.669, 0.908]	0.001	0.795	[0.682, 0.926]	0.003
35–39 X 2012–2013	0.749	[0.612, 0.916]	0.005	0.765	[0.626, 0.936]	0.009
40–44 X 1997–2000	0.975	[0.802, 1.185]	0.796	0.983	[0.809, 1.196]	0.866
40–44 X 2001–2003	0.788	[0.629, 0.987]	0.038	0.795	[0.635, 0.997]	0.047
40–44 X 2004–2007	0.704	[0.577, 0.858]	0.001	0.732	[0.600, 0.892]	0.002
40–44 X 2008–2011	0.698	[0.564, 0.865]	0.001	0.719	[0.580, 0.891]	0.003
40–44 X 2012–2013	0.583	[0.435, 0.781]	< 0.001	0.603	[0.450, 0.808]	0.001
45–49 X 1997–2000	0.658	[0.472, 0.916]	0.013	0.657	[0.471, 0.917]	0.013
45–49 X 2001–2003	0.484	[0.324, 0.722]	< 0.001	0.492	[0.329, 0.735]	0.001
45–49 X 2004–2007	0.227	[0.146, 0.352]	< 0.001	0.232	[0.149, 0.361]	< 0.001
45–49 X 2008–2011	0.279	[0.180, 0.432]	< 0.001	0.298	[0.192, 0.461]	< 0.001
45–49 X 2012–2013	0.107	[0.043, 0.266]	< 0.001	0.091	[0.033, 0.250]	< 0.001
Nationality						
South African				1.000	[1.000, 1.000]	.
Mozambican				1.325	[1.229, 1.429]	< 0.001
Mother's age X nationality						
20–24 X Mozambican				1.285	[1.191, 1.386]	< 0.001
25–29 X Mozambican				1.306	[1.207, 1.414]	< 0.001
30–34 X Mozambican				1.286	[1.182, 1.401]	< 0.001
35–39 X Mozambican				1.245	[1.130, 1.372]	< 0.001
40–44 X Mozambican				1.633	[1.430, 1.866]	< 0.001
45–49 X Mozambican				3.059	[2.360, 3.964]	< 0.001
Time period X nationality						
1997–2000 X Mozambican				0.881	[0.812, 0.955]	0.002
2001–2003 X Mozambican				0.859	[0.785, 0.940]	0.001
2004–2007 X Mozambican				0.806	[0.744, 0.873]	< 0.001
2008–2011 X Mozambican				0.661	[0.606, 0.720]	< 0.001
2012–2013 X Mozambican				0.673	[0.602, 0.753]	< 0.001
	Parameter			Parameter		
σ^2^_female_	0.03			0.00003		
ρ	0.008			0.000009		
N	337737			337047		

Unit of analysis is person-year, with variables defined at the beginning of each person-year

**Fig. 3:**
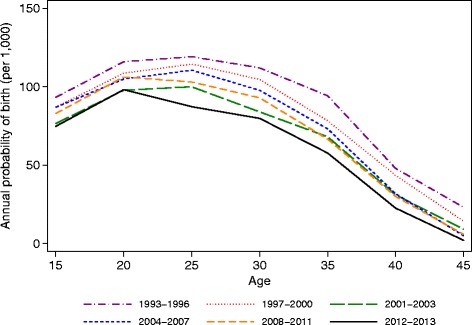
Annual probability of any birth by age group (15–49) and time period (1993–2013), Agincourt health and demographic surveillance system, South Africa. Multilevel model includes a random intercept for the individual

The odds ratios in model M1 describe the changes in a female’s probability of birth as a function of sex and age. Figure 3 shows the predicted probability of birth by age over time associated with these odds ratios. In 2001–2003, there was an overall decline in the probability of birth compared to 1993–1997 (ages 25–29 *p* <0.001). The probability of birth then increased in 2004–2007 relative to 2001–2003 (ages 25–29 *p* = 0.0170) but remained lower than the earliest time period (ages 25–29 *p* = 0.0480). The probability of birth decreased in the latest time period relative to 2008–2011 (ages 25–29 *p* = 0.002). The peak age also shifted in the last time period from ages 25–29 to ages 20–24 (*p* = 0.047).

### Fertility patterns by nationality

To examine differentials in fertility between nationality groups, we estimated the model M1 including a nationality covariate. Interactions between nationality and age (*p* < 0.001), and nationality and time (*p* < 0.001) significantly improved model fit and resulted in the final model. Estimation results are presented in model M2 (Table 2).

**Fig. 4:**
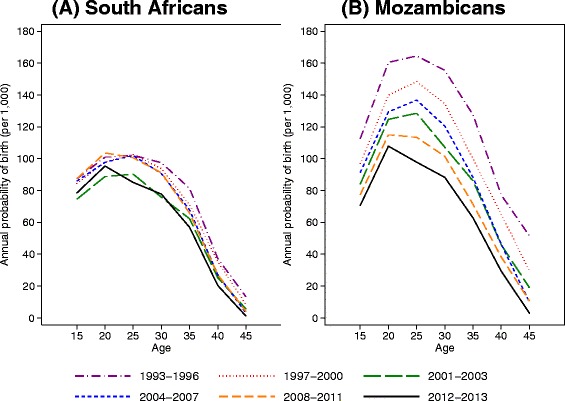
Annual probability of any birth by nationality (Panel (**a**) South African and Panel (**b**) Mozambican), age group (15–49), and time period (1993–2013), Agincourt health and demographic surveillance system, South Africa. Multilevel model includes a random intercept for the individual

For South Africans, in Fig. 4(a) fertility declined significantly across all age groups in 2001–2003 relative to 1993–1996. Fertility increased in 2004–2007 to similar levels as in 1993–1996 for ages 15–34 and remained high in 2008–2011. In the latest time period, fertility declined amongst ages 25–34 (*p* = 0.002; *p* = 0.012) and ages 40–49 (*p* = 0.044; *p* = 0.015).

For Mozambicans, in Fig. 4(b) fertility declined significantly across all age groups for each time period from 1997–2003. Fertility remained relatively stable from 2004–2007 relative to 2001–2003 and then declined in 2008–2011 among ages 15–39. Fertility continued to decline in 2012–2013 for ages 25–29 and 40–49.

### Fertility patterns by household socioeconomic status

To examine variations in fertility across household socioeconomic status, we estimated the model M1 (from Table 2) including tertiles of SES using the imputed SES values and restricted to years 2001–2013. Interactions between SES and age (*p* = 0.604), and SES and time (*p* = 0.979) were not significant. Estimation results are presented in model M3 (Table 3). There was an inverse gradient with SES, where the probability of birth declined as SES increased. This gradient held across all levels, with significant declines between the middle compared to the low SES tertiles (*p* < 0.001) and between the high compared to the middle SES tertiles (*p* < 0 .001).

**Table 3:** Logistic regression on birth by age, time, nationality, and household socio-economic status females ages 15–49, Agincourt health and demographic surveillance system, South Africa

	Model M3			Model M4		
	Odds ratio	95% CI	*p*-value	Odds ratio	95% CI	*p*-value
Mother's age						
15–19	1.000	[1.000, 1.000]	.	1.000	[1.000, 1.000]	.
20–24	1.302	[1.181, 1.436]	< 0.001	1.241	[1.119, 1.375]	< 0.001
25–29	1.356	[1.226, 1.5]	< 0.001	1.270	[1.141, 1.413]	< 0.001
30–34	1.123	[1.005, 1.255]	0.04	1.037	[0.922, 1.167]	0.542
35–39	0.890	[0.787, 1.007]	0.064	0.831	[0.729, 0.948]	0.006
40–44	0.395	[0.33, 0.473]	< 0.001	0.331	[0.272, 0.403]	< 0.001
45–49	0.112	[0.079, 0.157]	< 0.001	0.070	[0.046, 0.106]	< 0.001
Time period						
2001–2003	1.000	[1.000, 1.000]	.	1.000	[1.000, 1.000]	.
2004–2007	1.177	[1.077, 1.285]	< 0.001	1.183	[1.078, 1.299]	< 0.001
2008–2011	1.138	[1.031, 1.257]	0.011	1.207	[1.086, 1.34]	< 0.001
2012–2013	1.038	[0.907, 1.189]	0.588	1.121	[0.97, 1.294]	0.121
Mother's age X time period						
20–24 X 2004–2007	0.948	[0.837, 1.073]	0.397	0.955	[0.843, 1.082]	0.472
20–24 X 2008–2011	1.025	[0.895, 1.174]	0.724	1.016	[0.887, 1.164]	0.821
20–24 X 2012–2013	1.060	[0.885, 1.271]	0.526	1.045	[0.872, 1.253]	0.634
25–29 X 2004–2007	0.960	[0.843, 1.093]	0.535	0.965	[0.848, 1.099]	0.593
25–29 X 2008–2011	0.942	[0.817, 1.087]	0.414	0.940	[0.815, 1.085]	0.397
25–29 X 2012–2013	0.881	[0.728, 1.067]	0.194	0.872	[0.72, 1.056]	0.16
30–34 X 2004–2007	1.032	[0.896, 1.189]	0.663	1.043	[0.905, 1.202]	0.561
30–34 X 2008–2011	1.014	[0.867, 1.185]	0.861	1.018	[0.871, 1.19]	0.824
30–34 X 2012–2013	0.962	[0.782, 1.184]	0.716	0.954	[0.775, 1.175]	0.659
35–39 X 2004–2007	0.946	[0.807, 1.109]	0.492	0.941	[0.803, 1.104]	0.457
35–39 X 2008–2011	0.925	[0.775, 1.105]	0.389	0.922	[0.772, 1.102]	0.373
35–39 X 2012–2013	0.912	[0.718, 1.159]	0.453	0.903	[0.711, 1.147]	0.405
40–44 X 2004–2007	0.862	[0.683, 1.088]	0.211	0.884	[0.701, 1.117]	0.302
40–44 X 2008–2011	0.842	[0.651, 1.09]	0.193	0.857	[0.662, 1.11]	0.243
40–44 X 2012–2013	0.761	[0.533, 1.085]	0.131	0.766	[0.537, 1.094]	0.143
45–49 X 2004–2007	0.467	[0.279, 0.783]	0.004	0.467	[0.278, 0.784]	0.004
45–49 X 2008–2011	0.530	[0.305, 0.92]	0.024	0.560	[0.322, 0.973]	0.04
45–49 X 2012–2013	0.124	[0.03, 0.521]	0.004	0.126	[0.03, 0.528]	0.005
SES tertiles						
Low	1.000	[1.000, 1.000]	.	1.000	[1.000, 1.000]	.
Middle	0.862	[0.816, 0.911]	< 0.001	0.910	[0.859, 0.965]	0.003
High	0.778	[0.74, 0.818]	< 0.001	0.839	[0.795, 0.886]	< 0.001
Nationality						
South African				1.000	[1.000, 1.000]	.
Mozambican				1.152	[1.043, 1.271]	0.005
Mother's age X nationality						
20–24 X Mozambican				1.169	[1.054, 1.297]	0.003
25–29 X Mozambican				1.228	[1.101, 1.369]	< 0.001
30–34 X Mozambican				1.262	[1.121, 1.42]	< 0.001
35–39 X Mozambican				1.236	[1.081, 1.413]	0.002
40–44 X Mozambican				1.593	[1.316, 1.928]	< 0.001
45–49 X Mozambican				2.963	[1.91, 4.594]	< 0.001
Time period X nationality						
2004–2007 X Mozambican				0.943	[0.859, 1.034]	0.211
2008–2011 X Mozambican				0.781	[0.705, 0.865]	< 0.001
2012–2013 X Mozambican				0.749	[0.655, 0.857]	< 0.001
	Parameter			Parameter		
σ^2^_female_	8.87444E-06			5.08999E-06		
ρ	2.70E-06			1.55E-06		
N	187742			187635		

Household SES includes 5 imputations based on partial mean matching. Unit of analysis is person-year, with variables defined at the beginning of each person-year

Finally, we jointly modeled nationality and SES using the imputed SES values and restricted to years 2001–2013. We estimated the model M2 (from Table 2) along with SES tertiles. Interactions between nationality and SES were not significant (*p* = 0.836). Estimation results are presented in model M4 (Table 3). The overall age and time patterns from 2001–2013 (SES measurement began in 2001) between South Africans and Mozambicans remained similar to those in model M2. The SES gradient was attenuated but remained once controlling for nationality, with significant declines between the middle compared to the low SES tertiles (*p* = 0.003) and between the high compared to the middle SES tertiles (*p* = 0.003).

## Discussion

Several factors may explain these fertility trends, such as the HIV epidemic at its peak in 2004–2007. This may suggest that women were practicing replacement with elevated child mortality. Additionally, ART became available starting in 2007 and more widely in 2009 [28], which may help to explain the resumption of fertility decline in the latest time periods. Recent improved infant and child survival [27], likely the result of widespread take-up of prevention of mother-to-child transmission programs [34] may also help explain these findings. Further research is needed to explore the mechanisms and determinants of these observed fertility patterns.

The stall in fertility decline in this population from 2004–2007 has been documented by Ibisomi et al. [5] who found that this stall was most prominent in the South African population, who saw an increase in fertility. Our study corroborates their findings and further extends them, showing that the South African population’s fertility remained largely stable through 2013, with some decline in the last period, while Mozambican fertility continued to decline. However, Mozambican fertility remained substantially higher than South African fertility throughout this period, and the continued decline in Mozambican fertility through 2013 still did not achieve the level of fertility in the South African population. The Mozambican fertility decline through 2013 suggests that the fertility levels of the Mozambican population was in the process of equilibrating with the host population’s fertility throughout this period, often referred to as immigrant fertility assimilation.

The probability of a live birth declined with higher levels of SES, which is consistent with findings from other studies in sub-Saharan Africa using similar wealth indices. Adebowale et al. [35] suggests this effect may be due to differential access to family planning services in Malawi. Muhoza et al. [10] found differentials across SES and desired family size and excess fertility varied substantially across countries, while Dias, de Oliveira [36] found that community-level SES was a stronger predictor of fertility, with women in communities with higher levels of SES having lower fertility. Our findings demonstrate the differentials across SES seen in a poor, rural South African community for over a decade, which we are able to demonstrate with models that follow wealth trajectories for households rather than cross-sectional assessments at wide intervals. Neither time nor nationality had significant interactions with wealth tertiles, indicating that the effect of SES is independent of these other factors affecting fertility.

We acknowledge several strengths and limitations. We used a robust dataset with careful tracking of vital events over a long period of time. The site uses extensive training and quality control procedures to ensure high-quality data [37]. Because vital events are updated every year, births missed in 1 year are usually captured in the following year, since the missed individuals do not appear on pre-populated household rosters. Hence, completeness of recording of births into the Agincourt HDSS is very high despite some underreporting of births that end up as neonatal deaths between census rounds. However, data are from a geographically defined region in rural South Africa and this potentially limits the generalizability of our findings. In addition, the asset indices based on household assets that we use in this study are by no means the only way to measure SES. Since our asset indices do not include other factors associated with SES such as education, our findings may provide only a partial view of the multifaceted social patterning of the fertility transition in our study population. Our analyses used a unified, statistical framework, permitting detailed hypothesis testing of changes and variation in fertility trends. Since our analysis was focused on overall fertility trends, further studies are needed including important individual-level fertility predictors and linking women’s fertility with household-level mortality in order to understand the mechanisms driving overall fertility patterns in the population. Imputation of missing household SES values represents a methodological improvement over previous approaches such as carrying forward the most recent observation. Finally, the study setting has high HIV prevalence with a relatively late rollout of ART, allowing a careful examination of how patterns changed as the HIV epidemic unfolded. However, individual-level data on HIV and ART status each year are not available, limiting our analysis to time period associations with the two factors.

Our findings of overall declining fertility in 2013 raise the importance of family planning issues. Since transmission of HIV remains a major concern, notably among adolescents and young adults, it is increasingly important to ensure effective discussion and provision of family planning in the course of antenatal attendances and after delivery. Access to sound and accessible family planning advice, linked with personal protection against HIV infection, are vital and support the importance of integrated family planning and reproductive health services, especially given the growing numbers on long-term ART [38]. Particularly for adolescents and young adults who face high rates of HIV acquisition after graduating from secondary school, this reinforces the importance of adolescent-friendly health care and integrated family planning and reproductive health services [39].

## Conclusions

We summarized trends in fertility over time and age, and examined differences in fertility patterns by social groups. Overall fertility declined in 2001–2003, increased in 2004–2011, and then declined in 2012–2013. South Africans showed a similar pattern to the overall trends. Mozambicans showed a different pattern, with strong declines prior to 2003 before stalling during 2004–2007, and then continued fertility decline afterwards. There was an inverse gradient between fertility levels and household SES, with those in lower SES tertiles having higher levels of fertility. The gradient did not vary by time or nationality. Overall at the population level, the fertility transition in rural South Africa shows a pattern of decline until the height of the HIV/AIDS pandemic, with a resulting stall until further decline in the context of ART rollout. This study provides new insights about how current fertility behaviors are affected by the evolving HIV epidemic and continued availability of ART over a long period of follow-up, and how fertility behaviors differ between South African and Mozambican populations living in the study area.

## References

[1] Moultrie T, Timaeus I (2003). The South African fertility decline: Evidence from two censuses and a Demographic and Health Survey. Popul Stud.

[2] Statistics South Africa. Mid-year population estimates. Pretoria, South Africa2014 Contract No.: Statistical release P0302.

[3] DHS. Moçambique Inquérito Demográfico e de Saúde. Calverton, Maryland: Ministerio da Saude (MISAU), Instituto Nacional de Estatística (INE) e ICF International (ICFI)2011.

[4] Garenne M, Tollman S, Collinson M, Kahn K (2007). Fertility trends and net reproduction in Agincourt, rural South Africa, 1992–2004. Scand J Public Health Suppl.

[5] Ibisomi L, Williams J, Collinson MA, Tollman S (2014). The stall in fertility decline in rural, northeast, South Africa: the contribution of a self-settled, Mozambican, refugee sub-population. Afr Popul Stud.

[6] Bongaarts J, Watkins SC (1996). Social interactions and contemporary fertility transitions. Popul Dev Rev.

[7] Casterline JB (2001). The pace of fertility transition: National patterns in the second half of the twentieth century. Popul Dev Rev.

[8] Shapiro D, Gebreselassie T. Fertility transition in sub-Saharan Africa: falling and stalling. Afr. Popul. Stud. 2009;23(1):1-23.

[9] Kuang B, Ross J, Madsen EL (2014). Defining motivational intensity of need for family planning in Africa. Afr J Reprod Health.

[10] Muhoza DN, Broekhuis A, Hooimeijer P (2014). Variations in desired family size and excess fertility in East Africa. Int J Popul Res.

[11] Ndahindwa V, Kamanzi C, Semakula M, Abalikumwe F, Hedt-Gauthier B, Thomson DR (2014). Determinants of fertility in Rwanda in the context of a fertility transition: a secondary analysis of the 2010 Demographic and Health Survey. Reprod Health.

[12] Burger RP, Burger R, Rossouw L (2012). The fertility transition in South Africa: A retrospective panel data analysis. Dev South Afr.

[13] Williams J, Ibisomi L, Sartorius B, Kahn K, Collinson M, Tollman S (2013). Convergence in fertility of South Africans and Mozambicans in rural South Africa, 1993–2009. Glob Health Action.

[14] Magadi MA, Agwanda AO (2010). Investigating the association between HIV/AIDS and recent fertility patterns in Kenya. Soc Sci Med.

[15] Carpenter LM, Nakiyingi JS, Ruberantwari A, Malamba SS, Kamali A, Whitworth JA (1997). Estimates of the impact of HIV infection on fertility in a rural Ugandan population cohort. Health Transit. Rev..

[16] Juhn C, Kalemli-Ozcan S, Turan B (2013). HIV and fertility in Africa: first evidence from population-based surveys. J Popul Econ.

[17] Westoff CF, Cross AR (2006). The stall in the fertility transition in Kenya.

[18] Terceira N, Gregson S, Zaba B, Mason P (2003). The contribution of HIV to fertility decline in rural Zimbabwe, 1985–2000. Popul Stud.

[19] Marlow HM, Maman S, Moodley D, Curtis S, McNaughton RL (2015). HIV status and postpartum contraceptive use in an antenatal population in Durban. S Afr Contracept.

[20] Cooper D, Harries J, Myer L, Orner P, Bracken H (2007). “Life is still going on”: Reproductive intentions among HIV-positive women and men in South Africa. Soc Sci Med.

[21] Maier M, Andia I, Emenyonu N, Guzman D, Kaida A, Pepper L (2009). Antiretroviral therapy is associated with increased fertility desire, but not pregnancy or live birth, among HIV+ women in an early HIV treatment program in rural Uganda. AIDS Behav.

[22] Myer L, Carter RJ, Katyal M, Toro P, El-Sadr WM, Abrams EJ (2010). Impact of antiretroviral therapy on incidence of pregnancy among HIV-infected women in Sub-Saharan Africa: a cohort study. PLoS Med.

[23] Myer L, Morroni C, Rebe K (2007). Prevalence and determinants of fertility intentions of HIV-infected women and men receiving antiretroviral therapy in South Africa. AIDS Patient Care STDs.

[24] Kahn K, Collinson MA, Gomez-Olive FX, Mokoena O, Twine R, Mee P (2012). Profile: Agincourt Health and Socio-demographic Surveillance System. Int J Epidemiol.

[25] Gómez-Olivé FX, Angotti N, Houle B, Klipstein-Grobusch K, Kabudula C, Menken J (2013). Prevalence of HIV among those 15 and older in rural South Africa. AIDS Care.

[26] Houle B, Clark S, Gomez-Olive F, Kahn K, Tollman S (2014). The unfolding counter-transition in rural South Africa: mortality and cause of death, 1994–2009. PLoS One.

[27] Houle B, Clark SJ, Kahn K, Tollman S, Yamin AE (2015). The impacts of maternal mortality and cause of death on children’s risk of dying in rural South Africa: evidence from a population based surveillance study (1992–2013). Reprod Health.

[28] Gómez-Olivé FX. Measuring, monitoring, investigating and responding to the HIV epidemic in Agincourt: development of an HIV research agenda.Presentation at Wits AIDS Research Symposium. 2009.

[29] INDEPTH. Measuring Health Equity in Small Areas: Findings from Demographic Surveillance Systems. United Kingdom: Ashgate Publishing Ltd; 2005.

[30] Gelman A, Hill J (2007). Data Analysis Using Regression and Multilevel/Hierarchical Models.

[31] Allison PD (1984). Event history analysis: Regression for longitudinal event data.

[32] Rubin D (1987). Multiple Imputation for Nonresponse in Surveys.

[33] StataCorp. Stata Statistical Software: Release 13. 2013.

[34] Barron P, Pillay Y, Doherty T, Sherman G, Jackson D, Bhardwaj S (2013). Eliminating mother-to-child HIV transmission in South Africa. Bull World Health Organ.

[35] Adebowale SA, Adedini SA, Ibisomi LD, Palamuleni ME (2014). Differential effect of wealth quintile on modern contraceptive use and fertility: evidence from Malawian women. BMC Women’s Health.

[36] Dias JG, de Oliveira IT (2015). Multilevel effects of wealth on women’s contraceptive use in Mozambique. PLoS One.

[37] Kahn K, Tollman SM, Collinson MA, Clark SJ, Twine R, Clark BD (2007). Research into health, population and social transitions in rural South Africa: data and methods of the Agincourt Health and Demographic Surveillance System. Scand. J. Public Health.

[38] Garenne M, Tollman S, Kahn K, Collins T, Ngwenya S (2001). Understanding Marital and Premarital Fertility in Rural South Africa. J South Afr Stud.

[39] Geary RS, Gómez-Olivé FX, Kahn K, Tollman S, Norris SA (2014). Barriers to and facilitators of the provision of a youth-friendly health services programme in rural South Africa. BMC Health Serv Res.

